# El coraje de la verdad en las políticas de la vida: mostrar las desigualdades ante la muerte

**DOI:** 10.1590/S0104-59702024000100043

**Published:** 2024-10-11

**Authors:** Anahi Sy

**Affiliations:** i Profesora investigadora, Instituto de Salud Colectiva/Universidad Nacional de Lanus, Consejo Nacional de Investigaciones Científicas y Técnicas. Ciudad Autónoma de Buenos Aires – Argentina anahisy@gmail.com

Didier Fassin se vale de la sociología, la historia, la economía, la política, la demografía, la epidemiología, la filosofía y la literatura para presentar la antropología a un público más amplio. Formado en medicina, sociología y antropología, es convocado a dar la conferencia inaugural de la Cátedra de Salud Pública, en el Collège de France, el 16 de enero de 2020. Este libro es la traducción del texto que resulta de dicha conferencia, donde plantea que su trayectoria de trabajo lo conduce a hablar desde una perspectiva antropológica. Le interesan las ventanas que esta disciplina abre al mundo.

En tal sentido, escapa de las fórmulas preestablecidas para mostrar las desigualdades de las vidas y abordar el tema en toda su complejidad y profundidad; propone que la antropología es también portadora de perspectivas políticas: si el mundo puede ser de otra forma – y por cierto lo ha sido en el pasado y lo es en otros lugares –, entonces el cambio siempre es posible y alimenta otras esperanzas (Fassin, 2022, p.64).

El centro de su reflexión es sobre el valor que le otorgamos a la vida humana en diferentes grupos sociales. Se pregunta sobre qué valor le otorgamos, en el sentido de *value*, y señala que desde las religiones se tiene una concepción ideal, mientras que desde las filosofías existe una interpretación normativa, y tanto una como otra poco dicen sobre la forma en que las sociedades tratan a los seres humanos. En términos de *worth*, se mide como “esperanza de vida al nacer”, categoría que tampoco habla sobre el valor de la vida.

El autor demuestra, con ejemplos, cómo la desigualdad en cuanto a la esperanza de vida es consecuencia de la acumulación de desigualdades en la sociedad. Presenta el caso de Estados Unidos, que tiene las erogaciones en salud por habitante más elevadas del mundo, más del doble que Francia, pero el índice de esperanza de vida sitúa ese país en el lugar 46, detrás de Cuba y Estonia. Estados Unidos ocupa el puesto 49 en mortalidad infantil, el 42 en mortalidad juvenil y el 51 en mortalidad materna. Estados Unidos es un país donde la décima parte de la población adulta no tiene cobertura médica, y eso tiene un impacto que hace retroceder los promedios nacionales. Si nos detenemos en los ingresos de la población masculina adulta, la esperanza de vida del 1% más rico es 15 años superior a la del 1% más pobre; en poco más de una década el 5% más favorecido ganó dos años de vida más que el 15% más modesto.

Su argumentación va contra la simplificación de plantear que las personas negras viven menos porque son las más pobres y tienen menores estudios (lo cual oculta las desigualdades sociales que padecen las minorías étnico raciales). Destaca que en los Estados Unidos se dejó atrás la esclavitud para “legalizar la segregación y donde el movimiento por los derechos civiles suscitó, por contrapartida, un proceso de encarcelamiento en masa” (Fassin, 2022, p.45).

La esperanza de vida muestra que existen disparidades considerables de longevidad al comparar la de los más ricos con la de los más pobres; 13 años en Francia y 15 en Estados Unidos. Hasta ahora la comparación fue entre varones, en Francia (y este dato es válido para países occidentales), las mujeres tienen una mortalidad menor que los varones, independientemente de su categoría socio profesional. Así, por ejemplo, las trabajadoras manuales viven más tiempo que los varones que ejercen altos cargos gerenciales. Esto a menudo se ha descripto como privilegio del sexo femenino. Aunque si bien las trabajadoras manuales tienen una esperanza de vida dos años superior a la de los varones que desempeñan funciones jerárquicas, su esperanza de vida sin incapacidad es 7 años inferior, consecuencia de las condiciones de trabajo desfavorables; la ventaja aparente no es más que un artificio.

Retomando la pregunta inicial, emerge el interrogante sobre qué es la vida. El autor recupera a Canguilhem al plantear la distinción entre “lo viviente y lo vivido”. Lo viviente caracteriza a todos los seres vivos, incluyendo animales, plantas y bacterias. Y lo vivido sería propio de los seres humanos, lo biográfico narrable. La diferencia entre varones y mujeres hay que pensarla también en tales términos: las mujeres viven más que los varones ¿en qué condiciones?

Concluye que “la esperanza de vida no informa sobre calidad de vida, perceptible en términos de autonomía, emancipación o realización personal” (Fassin, 2022, p.50). Que las mujeres vivan más años que los varones no dice nada acerca de qué son sus vidas o, más específicamente, de qué hace de sus vidas la sociedad.

En tal sentido propone hablar de vida biológica y vida biográfica. “La esperanza de vida mide la extensión de la primera. La historia de vida relata la riqueza de la segunda. La desigualdad de las vidas sólo puede percibirse en reconocimiento de las dos” (Fassin, 2022, p.50). La paradoja de las mujeres muestra que una vida larga no alcanza para garantizar una vida buena. Y “la experiencia de los hombres afroamericanos recuerda que una vida desvalorizada termina por producir una vida arruinada”(p.51).

A propósito de un estudio sobre los elevados niveles de contagio de HIV entre varones que trabajan en una mina aurífera, muestra cómo la forma en que se organiza la vida y el trabajo en la mina favorece el elevado número de contagios. En tal sentido propone hablar de un “modo de producción” de la epidemia por las compañías mineras internacionales. Algo similar se señala para trabajadores agrícolas y podría pensarse para la industria agroalimentaria. En tal sentido, retoma el concepto de “necropolítica” de Achille Mbembe, que permite comprender cómo la vida en sentido biográfico deja su huella en la vida en el sentido biológico.

De este modo, la posibilidad de otra vida conduce a reconsiderar el sentido de la fórmula “esperanza de vida”, que dice cuántos años podemos esperar vivir, por “qué podemos esperar de la vida”. Fassin lúcidamente señala que hablar sobre desigualdad de las vidas ya no es solo interrogarse sobre las disparidades de su duración, sino considerar las diferencias entre lo que son y lo que los individuos tienen derecho a esperar de ellas. Ya no se habla de cantidad, sino de calidad; tampoco de longevidad, sino de dignidad: “La desigualdad en las vidas no se refiere a una duración de presencia en el mundo, sino a un ser en el mundo” (Fassin, 2022, p.57).

Así, devela cómo las desigualdades sociales ante la muerte se inscriben en los cuerpos y de qué modo podemos ir de la medición de las disparidades en la distribución de la muerte al examen de las inequidades conferidas a la vida y en la vida. Desde el culturalismo y moralismo se tiende a omitir o a perder de vista las estructuras sociales que producen las desigualdades y las políticas que muchas veces las agravan.

Plantea que la desigualdad es ante la vida misma y que, si bien las ciencias sociales no tienen una respuesta fácil a esta crisis, al menos pueden tener el “coraje de la verdad” en el sentido que le da Foucault (2010) en sus últimas conferencias en el Collège de France. Coraje que puede alimentar la inquietud que moviliza a la acción.

El libro está hermosamente narrado, intercalando las experiencias de campo de Fassin con su historia personal. En el último apartado del libro hay una entrevista realizada en 2021 por Sonia Budassi, periodista que además escribe la introducción al libro. Allí se retoman algunas cuestiones que Fassin plantea previamente. Señala que la política y la situación de Estados Unidos debería servir como ejemplo por sus consecuencias nefastas. Se trata del país más rico y se encuentra en el número 40 por su esperanza de vida en el nacimiento, detrás de Costa Rica, Chile, Perú y Colombia; tiene un gasto en salud un 54% más elevado que Japón y un 44% que Suiza, sin embargo, en su territorio, el promedio de la vida se sitúa, respectivamente, 5 y 6 años por debajo de estos países.

Propone hablar de *“*políticas de la vida”, en el sentido que Foucault da a biopolítica, esto es, la manera en que las sociedades tratan a las vidas humanas, y podríamos extenderlo a las no humanas. Propone avanzar sobre el concepto de biopoder, al referir a “biolegitimidad”, cuando aparece la primacía de lo biológico sobre lo biográfico. Esto está vinculado a la necesidad de reformular “expectativa de vida” como expectativas para la vida y la duración en el mundo por ser en el mundo.

Las estadísticas son lo que hacemos con ellas, en tal sentido puede permitir como impedir conocer las disparidades entre clases sociales (según se incorporen o no las categorías étnicas o raciales, por ejemplo).

Cuando se refiere a las desigualdades, se tiende a pensar en términos de desigualdad de las vidas biológicas, aunque es necesario pensar qué sucede con las desigualdades en las vidas biográficas, aquello que es no solo estar con vida, sino llevar una buena vida; deseada, soñada o por el contrario, lacerada, humillada, maltratada.

En tal sentido, si bien el libro se basa en la experiencia de trabajo etnográfico del autor en países europeos, las elaboraciones conceptuales desarrolladas, apelando a datos epidemiológicos para problematizarlos y reelaborarlos, resultan de enorme interés para el campo de la salud, si el interés se orienta a reducir las desigualdades y garantizar derechos. Si bien sus críticas y su mirada se encuentran en completa sintonía con lo que ya se viene planteando hace más de cinco décadas atrás desde la medicina social latinoamericana, la epidemiología crítica y el movimiento de salud colectiva; es necesario recrear múltiples formas de narrar y develar las desigualdades e inequidades hasta que ya no sea necesario hablar de ellas, porque ya no sean tales.

Así el libro se convierte en un recurso que muestra el valor diferencial que se otorga a la vida humana para, a partir de ello, reflexionar sobre la complejidad de las realidades latinoamericanas y elaborar novedosos modos de ver que movilicen a la acción en torno a las desigualdades en la vida y ante la muerte.
